# A degranulation assay using Vγ9Vδ2 T cells for the rapid diagnosis of familial hemophagocytic syndromes

**DOI:** 10.3389/fimmu.2024.1391967

**Published:** 2024-06-26

**Authors:** Olivia Jorisch-Mühlebach, Dina Pitts, Raphaela Tinner, Hong Ying Teh, Conrad Roelli, Seraina Prader, Stefano Vavassori, Jana Pachlopnik Schmid

**Affiliations:** ^1^ Division of Immunology and Children’s Research Center, University Children’s Hospital Zurich, Zurich, Switzerland; ^2^ Pediatric Immunology, University of Zurich, Zurich, Switzerland

**Keywords:** NK cell, K562, Vγ9Vδ2 T cell, gd T cell, HMBPP, degranulation assay, hemophagocytic lymphohistiocytosis, HLH

## Abstract

**Introduction:**

Hemophagocytic lymphohistiocytosis (HLH) is a life-threatening immune disorder characterized by uncontrolled lymphocyte and macrophage activation and a subsequent cytokine storm. The timely initiation of immunosuppressive treatment is crucial for survival.

**Methods:**

Here, we harnessed Vγ9Vδ2 T cell degranulation to develop a novel functional assay for the diagnosis of HLH. We compared the novel assay with the conventional natural killer (NK) cell stimulation method in terms of efficiency, specificity, and reliability. Our analysis involved 213 samples from 182 individuals, including 23 samples from 12 patients with degranulation deficiency (10 individuals with *UNC13D* deficiency, 1 with *STXBP2* deficiency, and 1 with *RAB27A* deficiency).

**Results:**

While both tests exhibited 100% sensitivity, the Vγ9Vδ2 T cell degranulation assay showed a superior specificity of 86.2% (n=70) compared to the NK cell degranulation assay, which achieved 78.9% specificity (n=213). The Vγ9Vδ2 T cell degranulation assay offered simpler technical requirements and reduced labor intensity, leading to decreased susceptibility to errors with faster processing times.

**Discussion:**

This efficiency stemmed from the sole requirement of dissolving (*E*)-4-hydroxy-3-methyl-but-2-enyl pyrophosphate (HMBPP) powder, contrasting with the intricate maintenance of K562 cells necessary for the NK cell degranulation assay. With its diminished susceptibility to errors, we anticipate that the assay will require fewer repetitions of analysis, rendering it particularly well-suited for testing infants.

**Conclusion:**

The Vγ9Vδ2 T cell degranulation assay is a user-friendly, efficient diagnostic tool for HLH. It offers greater specificity, reliability, and practicality than established methods. We believe that our present findings will facilitate the prompt, accurate diagnosis of HLH and thus enable rapid treatment and better patient outcomes.

## 1 Introduction

Hemophagocytic lymphohistiocytosis (HLH) is a rare but severe and life-threatening immune disorder; the prompt initiation of immunosuppressive treatment is therefore crucial for survival. HLH is characterized by the uncontrolled activation of lymphocytes and macrophages and the subsequent secretion of large amounts of pro-inflammatory cytokines (i.e. a “cytokine storm”) (1, 2).

Many different diseases can lead to HLH. Most forms of primary (or familial) HLH are caused by pathogenic genetic variants in the lymphocyte’s cytotoxic machinery (3, 4). Other genetic causes for HLH and macrophage activation syndrome (which are distinct from deficiencies in cytotoxicity) have been discovered recently (5). The so-called “acquired” forms of HLH can appear in a setting of severe infection, auto-inflammatory conditions, autoimmune disease, (lymphoid) malignancies or drug treatment (6).

The initial diagnosis of HLH or macrophage activation syndrome is based on a set of criteria (7, 8). The subsequent work-up seeks to identify a potential trigger and detect an underlying genetic predisposition to HLH. Prompt determination of the patient’s genetic predisposition to HLH significantly influences the subsequent care and the prognosis. While the clinician is awaiting a molecular diagnostic confirmation, rapid assays of protein expression and cell function have proven immensely beneficial in accelerating the diagnosis of primary HLH.

One of the best-established functional assays of degranulation deficiencies involves the detection of extracellular lysosomal associated membrane protein 1 (Lamp-1, CD107a); this protein is transiently exposed on the surface of cytotoxic cells after degranulation, which enables its measurement using flow cytometry (9). This assay is typically used to evaluate natural killer (NK) cell degranulation in response to the incubation of patient-derived peripheral blood mononuclear cells (PBMCs) with the erythroleukemia K562 cell line. The absence of the major histocompatibility complex class I antigen on K562 cells initiates cytotoxic granule delivery, which can be indirectly quantified by the measurement of CD107a expression on the NK cells. At present, this is the “gold standard” degranulation assay in diagnostic laboratories worldwide. It has outstanding sensitivity (nearly 100%) and slightly lower specificity (88%) (10, 11).

Although the K562-cell-line-based assay is well established, it has several limitations. Firstly, it is time-consuming and labor-intensive and requires a relatively large blood sample. Secondly, many external confounding factors (such as temperature changes during sample storage, and the K562 cells’ growth status, culture conditions, variations in K562 cell sourcing and passage) can introduce variability and reduce sensitivity. These variables can only be standardized in specialized laboratories with experience of performing NK cell degranulation assays (10, 11). Nevertheless, the introduction of the K562-based flow cytometry assay was a significant improvement over the previous cytotoxicity assays, which relied on radioactive chromium and thus required cumbersome radioactive safety monitoring procedures (12).

In order to overcome variability issues and make degranulation assays available to nonspecialized centers, other means of diagnosing deficiencies in cytotoxicity are needed. We therefore looked at whether the K562 cell line could be replaced by small antigenic molecules. When considering cells capable of cytotoxicity, the responsiveness of Vγ9Vδ2 T cells to phosphoantigens (pAgs) offers unique advantages (13). These include the homogeneity of the cell population’s response to stimulation and their robustness vis-à-vis long storage periods and cryopreservation. In some respects, yδ T cells expressing the Vγ9Vδ2 T-cell receptor (TCR) are similar to conventional cytotoxic αβ T lymphocytes because they form immune synapses and share the resulting signaling pathways (14). Vγ9Vδ2 T cells are not involved in major histocompatibility complex antigen presentation but rely on an alternative antigen presentation mechanism featuring butyrophillin 3 (BTN3) and BTN2A1 (15, 16). The Vγ9Vδ2 TCR combination is the most abundant among yδ T cells in the peripheral blood of healthy adults. Furthermore, the number of functional, perforin-producing cytotoxic Vγ9Vδ2 T cell is already high in the fetus and at birth relative to the less responsive and still immature NK cells (17).

HMBPP ((*E*)-4-hydroxy-3-methyl-but-2-enyl pyrophosphate, a pyro-phosphorylated metabolite of isoprenoid synthesis) is a pAg produced by eubacteria, cyanobacteria, plants, and apicomplexan protozoa. Its subsequent recognition by Vγ9Vδ2 T cells triggers a potent immune response (18).

The primary objective of the present study was to develop and evaluate a novel, rapid, easy-to-use functional assay based on Vγ9Vδ2 T cell degranulation, for the diagnosis of patients with suspected HLH. The novel assay was compared with the conventional K562 cell/NK cell co-incubation assay. We evaluated cell samples from patients with subsequently confirmed familial HLH and degranulation deficiency (hereafter referred to as the “fHLH group”) and from blood donors with no known degranulation deficiencies (hereafter referred to as the “control group”). The study’s secondary objective was to determine a threshold that could serve as a prospective cut-off for validation of the novel assay.

## 2 Materials and methods

This was an empirically designed prospective study. To assess degranulation capacity, the percentage of CD107a expression on the cell surface was analyzed after stimulation of either NK cells with K562 cells or Vγ9Vδ2 T cells with HMBPP. The effect of cryopreservation on isolated PBMCs and the effect of interleukin (IL)-2 prestimulation were also explored.

The blood sample analysis was approved by the institutional-review-board, Zürich, Switzerland (BASEC; reference: PB_2016_02280) and registered at Clinicaltrials.gov (NCT02735824).

### 2.1 Patient samples

**Figure 1:**
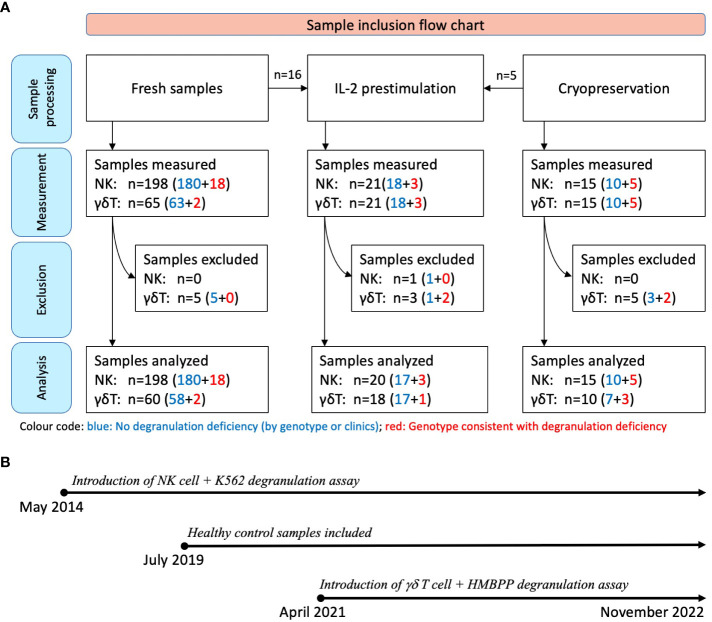
Sample inclusion flow chart and timeline. **(A)** All samples were initially assessed with at least one of the assays (K562- or HMBPP-induced expression of CD107a on NK cells or Vγ9Vδ2 T cells, respectively). Assays with fewer than 250 flow cytometry events were excluded (n=14). The sample donors were subsequently categorized as fHLH patients or controls after genetic testing or clinical follow-up: Blue: No degranulation deficiency; 190 samples from 170 individuals; with 18 of these being IL-2 prestimulated. Red: Genotype consistent with a degranulation deficiency; 23 samples from 12 patients with primary HLH [including homozygous or compound heterozygous variants in *UNC13D* (n=10), *STXBP2* (n=1), or *RAB27A* (n=1)]. Three of these samples were IL-2 prestimulated. **(B)** The NK cell degranulation assay was introduced in May 2014, while Vγ9Vδ2 T cell-based assay was introduced in April 2021. Blood samples were collected between May 2014 and November 2022, with healthy control samples collected from July 2019 onwards.

Between May 2014 and November 2022, a total of 213 consecutive samples from 182 individuals were considered for a functional degranulation assay in the diagnostic laboratory at University Children’s Hospital, Zurich (Zurich, Switzerland) (**Figure 1**). The cohort included patients having been referred for suspected HLH due to suggestive clinical symptoms or a positive family history and anonymous adult controls.

All the samples were tested with the conventional degranulation assay (i.e. with K562 target cells). Following the introduction of the novel HMBPP-stimulated degranulation assay in April 2021, all the samples thereafter were tested using both assays. Fifteen of the 213 samples were analyzed after cryopreservation, and 21 samples were analyzed after IL-2 prestimulation. To expand the sample and given that primary HLH is a very rare disease, we included 7 cryopreserved PBMC samples (5 samples from 2 patients with confirmed primary HLH and degranulation deficiency and 2 control samples) and 2 fresh blood samples (from a patient with confirmed primary HLH and degranulation deficiency) which were collected outside the main sampling period.

Samples were excluded if fewer than 250 events were recorded on flow cytometry analysis because this low count could not provide a reliable estimate of the proportion of NK cells or Vγ9Vδ2 T cells expressing CD107a.

### 2.2 Assay methods

#### 2.2.1 Sample acquisition, PBMC isolation, and cell stimulation

Venous whole blood samples were collected in heparin vials and sent to the laboratory within 24 hours for PBMC isolation (Ficoll (VWR International, 17–144-003) gradient centrifugation using SepMate™ technology (Stem Cell Technologies, 85450)). The extracted PBMCs were resuspended to a concentration of 2x10^6^ lymphocytes/mL, with 100 μL/well in Iscove’s modified Dulbecco’s medium (IMDM (Gibco, 12440–053)) supplemented with 10% FBS (Sigma, F7534), 1% amino acids (ThermoFisher, 11140–035), 1% antibiotics and antimycotics (Life Technologies, 15240–095), 1% GlutaMAX-I (ThermoFisher, 3505–0061) and 1% 100 mM sodium pyruvate (ThermoFisher, 11360–070). The PBMCs were stimulated separately with K562 cells and HMBPP + IL-2 (BioLegend, 589104) on a U-bottomed tissue culture plate. Each sample was run with a nonstimulated negative control (IMDM only). The plate was incubated for 2 hours at 37° in a 5% CO_2_ atmosphere before staining with specific mAbs for flow cytometry.

In parallel, blood samples collected in K_3_EDTA tubes were analyzed on a Sysmex XN analyzer for total lymphocyte counts.

#### 2.2.2 NK cell degranulation with K562 stimulation

K562 cells (ATCC, CCL-243) were cultivated in IMDM at 37°C in a 5% CO_2_ atmosphere. The culture medium was renewed every 3–5 days, and cell viability was evaluated under the microscope every 2–3 days. The assay used a 1:1 K562:PBMC ratio. After incubation on a U-bottomed culture plate, the cells were stained with a mixture of anti-CD107a PE (H4A3, BD Bioscience, 555801), anti-CD3 PerCP (SK7, BD Bioscience, 345766), and anti-CD56 APC (NCAM162, BD Bioscience, 341027).

#### 2.2.3 Vγ9Vδ2 T cell degranulation with HMBPP stimulation

For stimulation with HMBPP, 40 μL of diluted IL-2 (~100 IU/100µL), 1 μL of HMBPP (Echelon, I-M055 diluted 1mg/ml in Methanol) and 59 μL of IMDM were mixed. After the incubation, the cells were stained with a mixture of anti-CD107a PE (H4A3, BD Bioscience, 555801), anti-CD3 AmCyan (SK7, BD Bioscience, 339186), anti-CD56 APC (NCAM162, BD Bioscience, 341027), and anti-TCR Vγ9 FITC (REA470, Miltenyi Biotec, 130125204).

#### 2.2.4 Flow cytometry

Data were recorded with a FACS Canto II flow cytometer and analyzed with BD Diva software 8.0.1 (both from BD Bioscience). Surface CD107a expression was measured as a percentage (%CD107a) on gated CD3-CD56+ NK cells and CD3+TCRVγ9+ Vγ9Vδ2 T cells. Nonstimulated PBMCs were used to set the cut-off for a positive signal and to account for background noise. Gating strategies for the two assays are given in the **Supplementary Material**.

#### 2.2.5 IL-2 prestimulation of PBMCs

Assays that gave values close to or below the cut-off were repeated, in case pre-analytical or assay-derived problems had influenced the test results. A transient deficiency in NK cell degranulation with an undefined cause (such as *in vitro* damage to the NK cells) could also lead to values below the cut-off. An IL-2 prestimulation of the PBMCs for 6 to 15 days prior to K562 stimulation can help to correct false negative results, although it is time-consuming. The effect of IL-2 prestimulation was also tested for the Vγ9Vδ2 T cell degranulation assay prior to the HMBPP stimulation.

### 2.3 Statistical analysis

Statistical analyses were performed with GraphPad Prism software (version 9.2.0, GraphPad Software LLC, San Diego, CA). Spearman’s rank correlation coefficient was calculated as a guide to the strength of the relationship between two sets of assay results. The data’s distribution was evaluated with normal quantile-quantile (QQ) plots. A two-tailed Wilcoxon matched-pairs signed rank test was used to compare results for nonstimulated vs. stimulated cells. An unpaired Mann-Whitney test for nonparametric data was used to compare results from stimulated cells for the fHLH group vs. the control group. The specificity and sensitivity were calculated, and the two assays were compared in a contingency table. The threshold for statistical significance was set to p<0.05 in all tests.

## 3 Results

A total of 213 samples (**Table 1**) from 182 individuals (n=12 with confirmed primary HLH and degranulation deficiency; n=170 in whom these conditions were excluded or unknown) were assessed with at least one of the assays (K562- or HMBPP-induced expression of CD107a on NK cells or Vγ9Vδ2 T cells, respectively). Repeated samplings and sample analyses were included. Of those individuals presenting with primary HLH, we analyzed 23 samples in total (20 samples of 10 individuals with *UNC13D* deficiency; 1 sample of 1 individual with *STXBP2* deficiency; 2 samples of 1 individual with *RAB27A* deficiency, **Table 2**).

**Table 1:** Demographic and clinical characteristics of the individuals having provided samples.

	K562 assay	HMBPP assay
	n=213	n=80
Cryopreserved samples	15 (7.1%)	15 (18.8%)
Demographic characteristics:		
*male*	92 (43.2%)	30 (37.5%)
*female*	68 (31.9%)	15 (18.8%)
*unknown (anonymous controls)*	53 (24.9%)	35 (43.8%)
median age (range):	3.0 (0–56)	1.0 (0–44)
*< 1 months (mean ± SD) in days*	12 (± 8.2)	20 (± 7.2)
*1–12 months (mean ± SD) in months*	3.5 (± 2.8)	3.9 (± 3.3)
*1–5 years (mean ± SD)*	2.4 (± 1.4)	2.4 (± 1.3)
*6–10 years (mean ± SD)*	7.4 (± 1.4)	9.0 (± 0.8)
*11–18 years (mean ± SD)*	14 (± 2.7)	15 (± 2.3)
*adults (>18 years) (mean ± SD)*	39 (± 11)	38 (± 4.3)
*healthy adult controls, anonymous (n)*	53	35
Clinical characteristics:		
*fHLH (a genetically confirmed degranulation deficiency)*	23 (10.8%)	7 (8.8%)
*HLH-like disease/HLH without a degranulation deficiency (Prf, XLP1, XIAP, CD48, ZNFX1)*	15 (7.0%)	1 (1.3%)
*primary immunodeficiency without an HLH-like phenotype*	5 (2.4%)	2 (2.5%)
*malignancies*	9 (4.2%)	1 (1.3%)
*autoimmune/rheumatologic disease*	11 (5.2%)	2 (2.5%)
*infectious disease*	12 (5.6%)	4 (5.0%)
*PIMS/Kawasaki syndrome*	17 (8.0%)	3 (3.8%)
*metabolic disorder*	7 (3.3%)	2 (2.5%)
*hematologic disease*	9 (4.2%)	5 (6.3%)
*unknown*	51 (24.0%)	18 (22.5%)
*healthy controls, anonymous*	54 (25.4%)	35 (43.8%)
mean CRP (± SD); n	41 (± 65); n=86	48 (± 78); n=22

The degranulation assays were performed on fresh or cryopreserved samples from patients with suspected degranulation deficiencies and control individuals. SD, Standard deviation; fHLH, familial Hemophagocytic lymphohistiocytosis; PIMS, Pediatric Inflammatory Multisystem Syndrome; CRP, C-reactive protein.

**Table 2:** A total of 23 samples from 12 patients with a genetically confirmed degranulation impairment were collected.

	patients	samples
Total fHLH	n=12	n=23
*UNC13D*	10	20
*STXBP2*	1	1
*RAB27A*	1	2

Repeated samplings and sample analyses were included.

### 3.1 The NK cell degranulation assay with fresh blood

**Figure 2:**
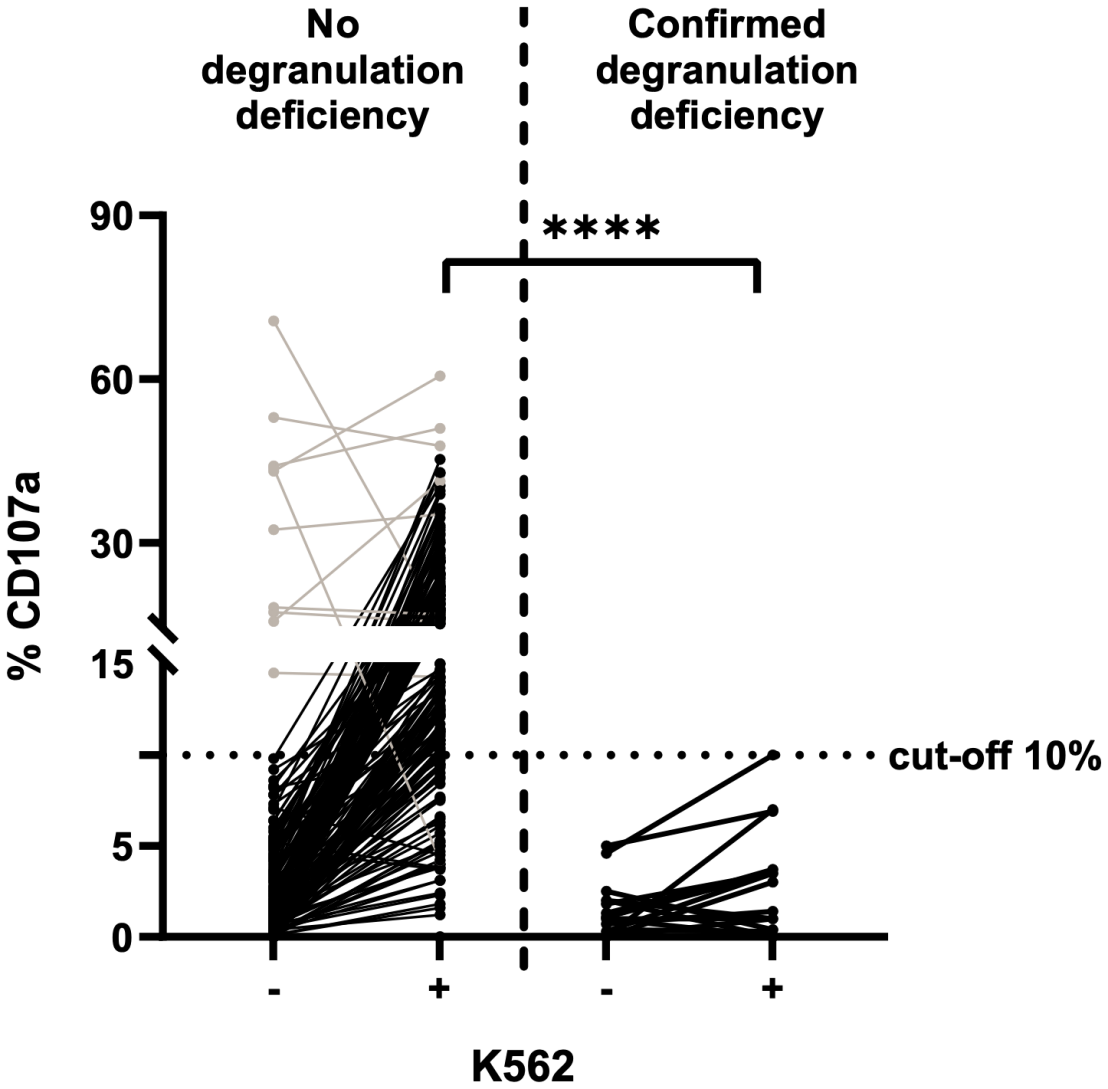
NK cell degranulation assay on fresh blood samples, using K562 cells for stimulation (n=198) and CD107a expression as a marker for degranulation. The cut-off for insufficient degranulation was 10%. Left: fresh blood samples from control individuals. Right: fresh blood samples from patients with a subsequently confirmed genetic deficiency in degranulation (fHLH). Values before (-) and after (+) stimulation with K562 cells are shown for both groups. Patients with a transient NK cell degranulation deficiency of unknown reason are included. Results for samples with a high degranulation rate prior to stimulation are shown in grey. The %CD107a values before vs. after stimulation differed significantly in the control group but not in the fHLH group (p<0.0001 and p=0.0289, respectively; two-tailed Wilcoxon matched-pairs signed rank test). After stimulation, the %CD107a differed significantly when comparing the fHLH group and the control group (****p<0.0001, Mann-Whitney test). The assay’s sensitivity was 100%, and the specificity was 78.3% (39 false positives and 141 true negatives).

The NK cell degranulation assay (based on incubation of K562 with PBMCs isolated from fresh blood samples) is the gold standard for the diagnosis of degranulation deficiencies. The cut-off for CD107a expression on NK cells is 10%. We performed the assay with 198 PBMC samples from fresh blood: 18 from fHLH patients and 180 from control individuals (**Figure 2**). In the control group, the proportion of CD107a expression (%CD107a) for NK cells ranged from 0.0 to 70.7% (mean ± standard deviation (SD) value of 3.9 ± 9.0%) before stimulation with K562 cells and from 0.0–60.6% (mean ± SD: 17.5 ± 10.6%) after incubation (p<0.0001). Most samples showed a low %CD107a (<10%) at baseline and displayed an elevation in CD107a expression upon stimulation with K562 cells. For unknown reasons, 10 samples showed an elevated %CD107a (>10%) before stimulation. We wondered whether the cells had already been stimulated in some way, although the clinical data from control individuals did not indicate any inflammatory conditions, and these elevated degranulation rates were not correlated with the donors’ serum level of C-reactive protein within ±2 days of sampling (**Table 1**).

The %CD107a with NK cells was lower in the fHLH group than in the control group, with a range of 0.0–5.0% (mean ± SD: 1.4 ± 1.5%) before stimulation and 0.0–10.0% (mean ± SD: 2.8 ± 2.8%) afterwards (p=0.0289). The difference in the stimulated %CD107a between the fHLH group and the control group was statistically significant (p<0.0001, Mann-Whitney test).

### 3.2 The NK cell degranulation assay with fresh and cryopreserved samples

**Figure 3:**
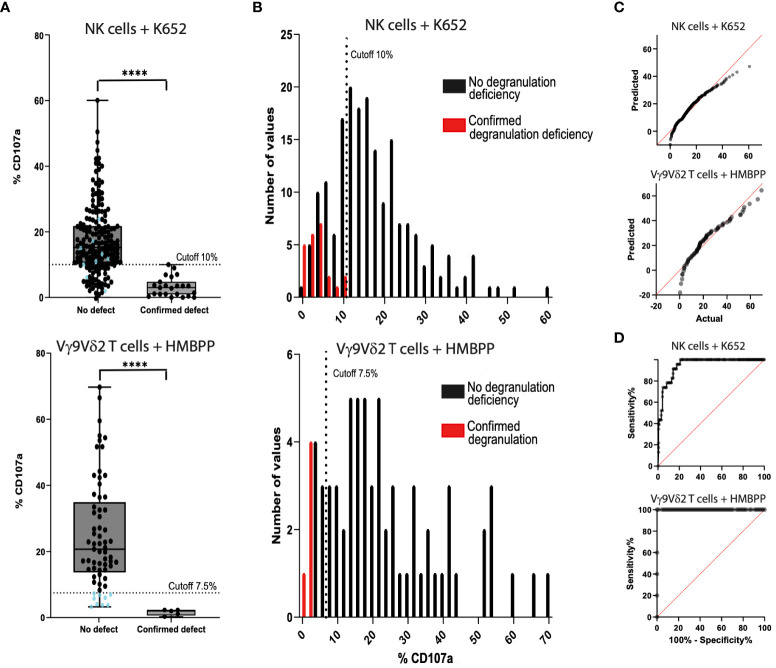
Comparison of the NK cell degranulation assay (n=213) and the Vγ9Vδ2 T cell degranulation assay (n=80) with fresh blood samples and cryopreserved PBMC samples. **(A)** Samples from control individuals (on the left of the graph) and samples from fHLH patients (on the right) were compared using K562-stimulated cells (top) and HMBPP-stimulated cells (bottom) (****p<0.0001 in a Mann-Whitney test). The %CD107a cut-off was set to 7.5% in the Vγ9Vδ2 T cell degranulation assay. There is a grey area in the readouts between 7.5% and 13.4% (the 25^th^ percentile of the control samples), where the assay has to be repeated. Blue dots: %CD107a values below the 7.5% cut-off analyzed with the Vγ9Vδ2 T cell degranulation assay (bottom) and the correlating results of the same samples with the NK cell degranulation assay (top). **(B)** The sensitivity was 100% for both tests. The specificity was 78.9% (40 false positives and 150 true negatives) after stimulation with K562 cells (top) and 86.2% (9 false positives and 56 true negatives) after HMBPP stimulation (bottom). **(C)** QQ plots for the PBMCs after stimulation with K562 cells (top) or with HMBPP (bottom). **(D)** The area under the ROC curve was 0.948 (p<0.0001) for K562-stimulated cells (top) and 1.0 (p<0.0002) for HMBPP-stimulated cells (bottom).

The NK cell degranulation assay with the K562 cell line was also performed with the additional cryopreserved PBMC samples (n=213) (**Figure 3**). After stimulation, the %CD107a ranged from 0.0% to 60.6% (mean ± SD: 17.6 ± 10.6%) with PBMCs from the control group (n=190) and from 0.0% to 10.0% (mean ± SD: 3.2 ± 2.9%) with PBMC samples from the fHLH group (n=23). The QQ plot showed that the data were not normally distributed. After K562 stimulation, the %CD107a differed significantly when comparing the fHLH and control groups (p<0.0001, Mann-Whitney test). The sensitivity was 100% and the specificity was 78.9% (40 false positives and 150 true negatives, **Table 3**). In a receiver operating characteristic (ROC) curve analysis, the area under the curve was 0.948 (p<0.0001).

**Table 3:** Contingency table for the evaluation of the sensitivity and specificity of the NK cell degranulation assay and Vγ9Vδ2 T cell degranulation assay.

NK cell degranulation assay (n=213, 0 exclusions)	Confirmed primary HLH	No primary HLH
Positive test (degranulation defect)	23 (10.8%)	40 (18.8%)
Negative test (no degranulation defect)	0 (0.0%)	150 (70.4%)
	Sensitivity: 100%	Specificity: 78.9%

Vγ9Vδ2 T cell degranulation assay (n=80, 10 exclusions)	Confirmed primary HLH	No primary HLH
Positive test (degranulation defect)	5 (7.1%)	9 (12.9%)
Negative test (no degranulation defect)	0 (0.0%)	56 (80.0%)
	Sensitivity: 100%	Specificity: 86.2%

Genetic testing was used as the reference in the diagnosis of HLH, and the clinical course was used as the reference for healthy control individuals. The sensitivity was 100% for both tests. The specificity was 78.9% (n=213) for the NK cell degranulation assay and 86.2% (n=80, 10 exclusions) for the Vγ9Vδ2 T cell degranulation assay.

### 3.3 The Vγ9Vδ2 T cell degranulation assay using fresh and cryopreserved samples

After HMBPP-dependent induction of freshly isolated or cryopreserved PBMC samples, the %CD107a for Vγ9Vδ2 T cells ranged from 3.3% to 66.0% (mean ± SD: 24.9 ± 16.3%) in the control group (n=80, 10 samples excluded, **Figure 3**) and from 0.3% to 2.0% (mean ± SD: 1.6 ± 0.9%) in the fHLH group (p<0.0001, Mann-Whitney test). The QQ plot showed that the data were not normally distributed. With a cut-off of 7.5%, the sensitivity was 100% and the specificity was 86.2% (9 false positives and 56 true negatives, **Table 3**). In a ROC curve analysis, the area under the curve was 1.0 (p<0.0002).

### 3.4 Comparison of the NK cell degranulation assay and Vγ9Vδ2 T cell degranulation assay

By drawing up a contingency table, we confirmed that the HMBPP induced Vγ9Vδ2 T cell degranulation had a higher specificity (86.2%, n=80) than the K562 based NK cell degranulation assay (78.9%, n=213). The sensitivity was 100% for both assays (**Table 3**). The proportion of samples excluded due to an event count <250 was higher in the Vγ9Vδ2 T cell degranulation assay (12.5%) than in the NK cell degranulation assay (0%). The positive predictive value was 36.5% for the NK cell degranulation assay and 35.7% for the Vγ9Vδ2 T cell degranulation assay. The negative predictive value was 100% for both assays.

**Figure 4:**
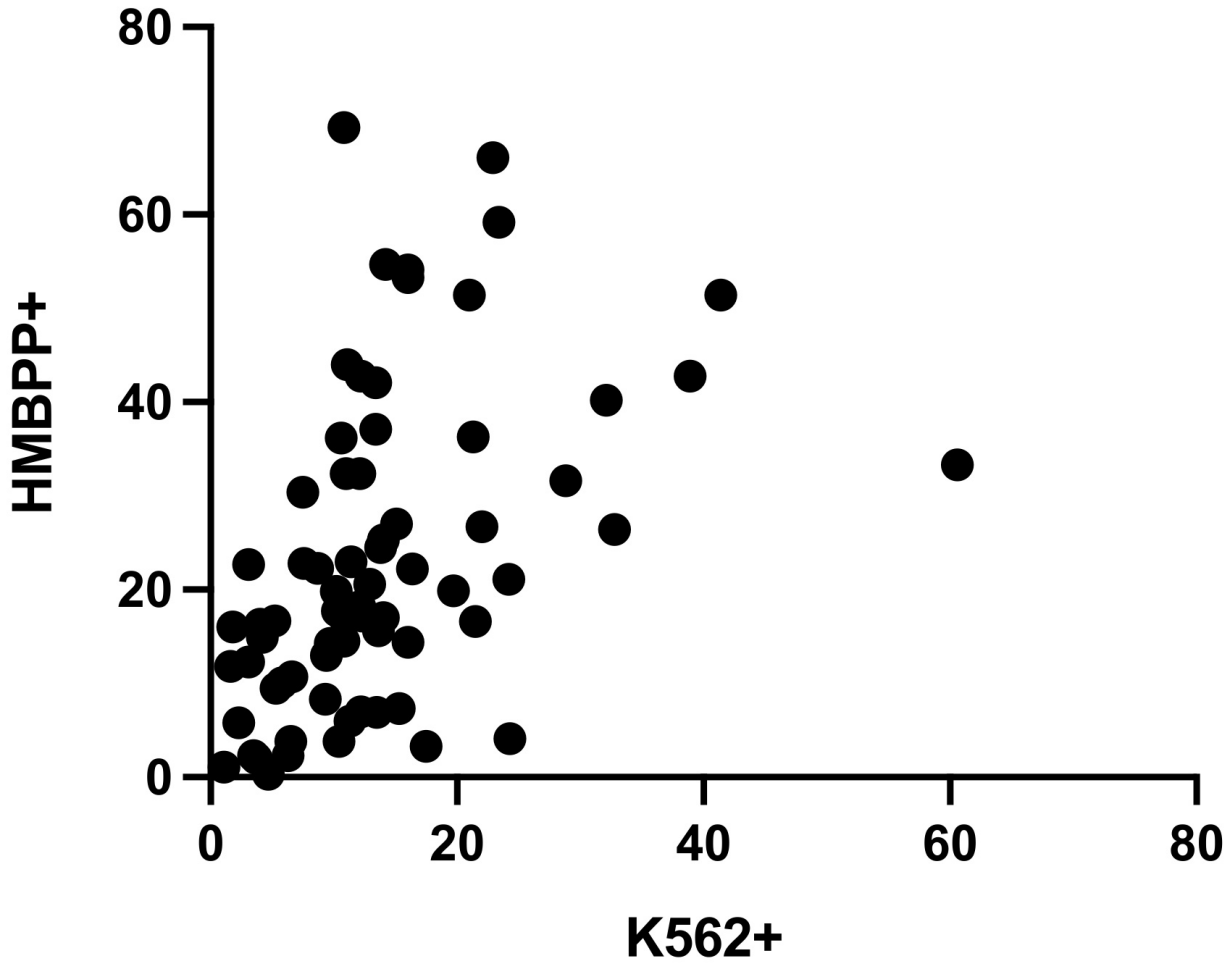
Spearman’s rank correlation coefficient for CD107a expression in the NK cell degranulation assay and the Vγ9Vδ2 T cell degranulation assay was r=0.546 (n=80, 10 exclusions, p<0.0001).

The Spearman’s rank correlation coefficient for samples tested with both the NK cell degranulation assay and the Vγ9Vδ2 T cell degranulation assay (n=80) was 0.546, after stimulation with K562 or HMBPP, respectively. Hence, the two datasets were not significantly correlated (p<0.0001) (**Figure 4**).

In our cohort, some individuals received treatment before blood sampling. To assess the impact of immunosuppression on assay outcomes, we compared treated and untreated individuals. Patients receiving corticosteroid treatment equivalent to Methylprednisolone ≥1 mg/kg were classified as immunosuppressed. However, our findings did not indicate a significant influence of this treatment on assay outcomes (data not shown).

The limited frequency of both NK cells and Vγ9Vδ2 T cells poses a challenge, particularly in scenarios with restricted blood sample volumes, often experienced in infants. **Table 4** illustrates the percentage distribution of NK cells and Vγ9Vδ2 T cells among total lymphocytes in infants aged ≤ 1 year. Although larger variances were noted in the percentages of Vγ9Vδ2 T cells, the percentages of NK cells did not significantly differ from those of Vγ9Vδ2 T cells among lymphocytes.

**Table 4:** Percentage distribution of NK cells and Vγ9Vδ2 T cells among total lymphocytes in infants ≤ 1 year.

	NK cells in % of lymphocytes	Vγ9Vδ2 T cells in % of lymphocytes
	n=15	n=14
Minimum	0.8	0.3
10% Percentile	1.6	0.3
90% Percentile	8.6	34.9
Maximum	9.6	67.2
Median	3.1	0.5
Mean	4.2	5.4
Unpaired t test	P value = 0.79	

The data represents NK cell and Vγ9Vδ2 T cell percentages determined after PBMC isolation, while total lymphocyte counts were determined from whole blood samples.

### 3.5 IL-2 prestimulation prior to degranulation assays

**Figure 5:**
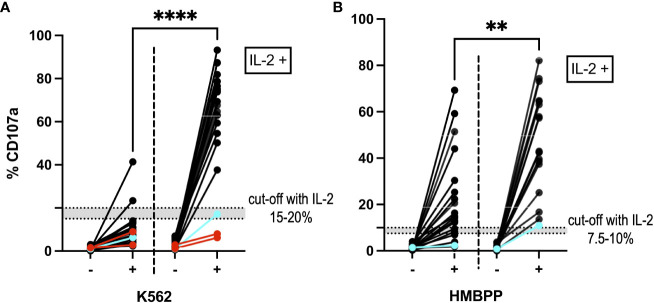
NK cells and Vγ9Vδ2 T cells after IL-2 prestimulation. **(A)** The NK cell degranulation assay and **(B)** the Vγ9Vδ2 T cell degranulation assay showed higher %CD107a values after incubation with IL-2 (on the right-hand side of each panel; n=21). The graph shows the %CD107a readouts before (-) and after (+) stimulation with K562 cells and HMBPP, respectively. By way of a comparison, the PBMC degranulation before IL-2 prestimulation is also shown on the left of each graph. ****p<0.0001 and **p=0.0014 in a Mann-Whitney test. Blue: A cryopreserved PBMC sample from a patient with a degranulation deficiency (*RAB27A* deficiency) showed an increase in %CD107a after IL-2 prestimulation. Red: Two cryopreserved PBMC samples from a patient with *UNC13D* mutations gave a %CD107a value below 10%, even after IL-2 prestimulation. In the HMBPP assay, these samples met our exclusion criteria and so are not shown.

Incubation of PBMCs with IL-2 for 6 to 15 days before incubation with the K562 cell line or stimulation with HMBPP led to an increase in degranulation (p<0.0001; two-tailed Wilcoxon matched-pairs signed rank test). The difference in %CD107a before and after IL-2 incubation was statistically significant (p<0.0001 for K562-stimulated cells and p=0.0014 for HMBPP-stimulated cells; Mann-Whitney test). Our suggested cut-off after IL-2 incubation was 15–20% for the K562-induced NK cell degranulation assay and 7.5–10% for the HMBPP-induced Vγ9Vδ2 T cell degranulation assay (**Figure 5**). Some samples from patients with primary HLH (e.g. *RAB27A* deficiency, also known as Griscelli syndrome) gave normal degranulation results after IL-2 prestimulation.

## 4 Discussion

A novel functional assay of CD107 expression on Vγ9Vδ2 T cells in response to *in vitro* stimulation with HMBPP proved to be a user-friendly, efficient means of diagnosing degranulation deficiencies. Relative to the established NK cell degranulation assay, the new assay showed a higher specificity (78.9% vs. 86.2%, respectively) and the same, high sensitivity (100%). Furthermore, the novel assay was more reliable, less labor-intensive, more rapid, and less susceptible to errors than the NK cell degranulation assay. Given its decreased vulnerability to errors, we expect that the Vγ9Vδ2 T cell degranulation will necessitate fewer repetitions of analysis, making it especially suitable for testing infants.

The new Vγ9Vδ2 T cell degranulation assay showed a lower cut-off than the conventional NK cell degranulation assay and also distinguished more clearly between positive results (for healthy individuals) and negative results (individuals with a degranulation deficiency). These advantages reduced the need to repeat the assay, which is a crucial advantage in an urgent medical situation. We set the %CD107a cut-off to 7.5%, with a grey area (in which the test should be repeated) between 7.5% and 13.4% (the 25^th^ percentile of the CD107a expression for samples from control individuals without a degranulation deficiency). We also showed that the assay could be performed on cryopreserved PMBCs and that it revealed an increase in degranulation capacity after incubation of the PBMCs with IL-2 (19). The latter feature could be used to check results close to the cut-off and thus further characterize the PBMCs’ degranulation capacity.

As reported by Bryceson et al. (2012) and by our group, the NK cell degranulation assay shows high intra-sample variability in the %CD107a; this is mainly due to factors that influence the quality of the K562 cells and the PBMC sample. The novel Vγ9Vδ2 T cell degranulation assay is less susceptible to this variation. Moreover, there is no need to culture a cell line. Nevertheless, the interpretation of the novel assay’s results might not be reliable if too few Vγ9Vδ2 T cells are counted by flow cytometry. Detection of CD107a upregulation in both Vγ9Vδ2 T cells and NK cells poses inherent challenges due to their low frequency, particularly in scenarios where blood sample volumes are limited, as is often the case with infants. Furthermore, we found that the event count in the Vγ9Vδ2 T cell degranulation assay was sometimes low for cryopreserved PBMC samples. It remains to be established whether the cell membrane and/or other components were damaged by freezing. Furthermore, the literature data on the mechanism of HMBPP’s activation of the Vγ9Vδ2 T cells are contradictory: some reports mention an intracellular mechanism, whereas others mention an extracellular mechanism involving a transmembrane protein from the butyrophilin family (20).

We tested both assays with freshly isolated and cryopreserved PBMC samples, which is consistent with routine clinical practice. Although the use of cryopreserved samples in the Vγ9Vδ2 T cell degranulation assay expanded the sample size, the number of samples from patients with fHLH remained small. Other study limitations included the lack of double-blind testing, the single-center study design, and the relative short sampling period.

In the future, larger multicenter cohort studies will be required to define a consistent %CD107a cut-off for the Vγ9Vδ2 T cell degranulation assay. Furthermore, the degranulation of cord blood samples and PBMCs from adults, adolescents and infants should be analyzed to investigate potential age-related differences in control individuals. Moreover, sources of error in the Vγ9Vδ2 T cell degranulation assay and reasons for false negative results should be determined.

It is thought that a significant proportion of patients with primary HLH are not diagnosed because of a lack of awareness of the condition or its resemblance to sepsis. The lack of an accurate diagnosis or timely treatment ultimately lead to severe organ failure or death. Hematopoietic stem cell transplantation (HSCT) has potential as a curative treatment for primary HLH (21, 22). Post-HSCT outcomes are notably better when patients are free from active disease at the time of transplantation. In families with primary HLH, the overall survival rate is better for a second affected child than for the index sibling (23–26). Newborn screening therefore has significant potential for improving the prognosis in cases of primary HLH. Most rare disease experts agree that DNA sequencing could be used to screen apparently healthy newborns for treatable genetic disorders (27). Before sequencing-based newborn screening programs can be implemented, we must be able to distinguish between disease-causing variants and benign variants of all the screened genes (28). The Vγ9Vδ2 T cell degranulation assay might be a valuable addition to a newborn screening program as a means of functionally validating a positive genetic test results. It might also constitute a rapid screening method for prompt treatment initiation in an emergency context. Lastly, the low volume of blood required for the assay increases its feasibility.

In conclusion, a novel Vγ9Vδ2 T cell degranulation assay with HMBPP stimulation is more specific and faster than the conventional NK cell degranulation assay. The novel assay is less sensitive to error, is less labor-intensive, and enables the rapid diagnosis of a degranulation deficiency. *In vitro* IL-2 prestimulation prior to the assay helps to classify samples close to the cut-off. This Vγ9Vδ2 T cell degranulation assay is a feasible, very sensitive method for detecting degranulation deficiencies in freshly isolated and cryopreserved PBMCs.

## Data availability statement

The raw data supporting the conclusions of this article will be made available by the authors, without undue reservation.

## Ethics statement

The studies involving humans were approved by institutional-review-board, Zürich, Switzerland. The studies were conducted in accordance with the local legislation and institutional requirements. Written informed consent for participation in this study was provided by the participants’ legal guardians/next of kin.

## Author contributions

OJ-M: Writing – original draft, Writing – review & editing, Investigation, Formal analysis, Methodology, Validation, Visualization. DP: Resources, Software, Investigation, Writing – review & editing, Writing – original draft, Visualization, Validation, Methodology, Formal analysis. RT: Writing – review & editing, Formal analysis, Writing – original draft. HYT: Writing – original draft, Writing – review & editing, Investigation, Formal analysis. CR: Writing – original draft, Investigation, Writing – review & editing, Formal analysis. SP: Writing – review & editing, Resources, Writing – original draft. SV: Writing – original draft, Writing – review & editing, Conceptualization, Methodology. JPS: Supervision, Writing – review & editing, Writing – original draft, Resources, Project administration, Funding acquisition.
