# Supplementary material for: A degranulation assay using Vγ9Vδ2 T cells for the rapid diagnosis of familial hemophagocytic syndromes
Source: Front Immunol. 2024 Jun 26;15:1391967. doi: 10.3389/fimmu.2024.1391967 (PMC11233720; doi:10.3389/fimmu.2024.1391967)
Supplement: Supplementary file 1 [file DataSheet_1.pdf]

Supplementary Material

A degranulation assay using Vγ9Vδ2 T cells for the rapid diagnosis of familial hemophagocytic syndromes

Olivia Jorisch-Mühlebach, Dina Pitts, Raphaela Tinner, Hong Ying Teh, Conrad Roelli, Seraina Prader, Stefano Vavassori, Jana Pachlopnik Schmid*

*** Correspondence:** Jana Pachlopnik Schmid: jana.pachlopnik@kispi.uzh.ch

1 Supplementary Data

1.1 Test methods

1.1.1 Gating strategy

1.1.1.1 The NK cell degranulation assay

To interpret the %CD107a expression of the stimulated NK cells, gates have to be set on the unstimulated PBMC sample, as listed below (**Figure 1**):

1) Gate lymphocytes
2) Gate singlets
3) Gate CD56+/CD3- NK cells
4) Set a cut-off for the CD107a gate on the unstimulated PBMC sample tube
5) Interpretation of the %CD107a expression on the stimulated PBMC sample tube (**Figure 2**)

1.1.1.2 The Vγ9Vδ2 T cell degranulation assay

To interpret the %CD107a expression of the stimulated Vγ9Vδ2 T cells, gates have to be set on the unstimulated PBMC sample tube, as listed below (**Figure 3**):

1) Gate lymphocytes
2) Gate singlets
3) Gate NK/T cells
4) Gate Vγ9Vδ2 T cells
5) Set a cut-off for the CD107a gate on the unstimulated PBMC sample tube
6) Interpretation of the %CD107a expression on the stimulated PBMC sample tube (**Figure 4**)

1.1.2 Standard operating procedures of the degranulation assay

The Standard operating procedures of the degranulation assays are available on request.

1.2 Outcomes of the degranulation assays with cryopreserved PBMCs

To increase the sample size, we tested cryopreserved samples of PBMCs from patients with genetically confirmed degranulation deficiencies in both assays (Figure 6). Both the NK cell and the Vγ9Vδ2 T cell degranulation assay provided reliable results with cryopreserved samples (n=15). All the samples from fHLH patients displayed values below the cut-off (sensitivity: 100%). The specificity was 90%, for the NK cell degranulation assay and 85.7% for the Vγ9Vδ2 T cell degranulation assay. We do not know why the samples of the control group had pathologic CD107a expression, as they came from anonymous donors.

2 Supplementary Figures

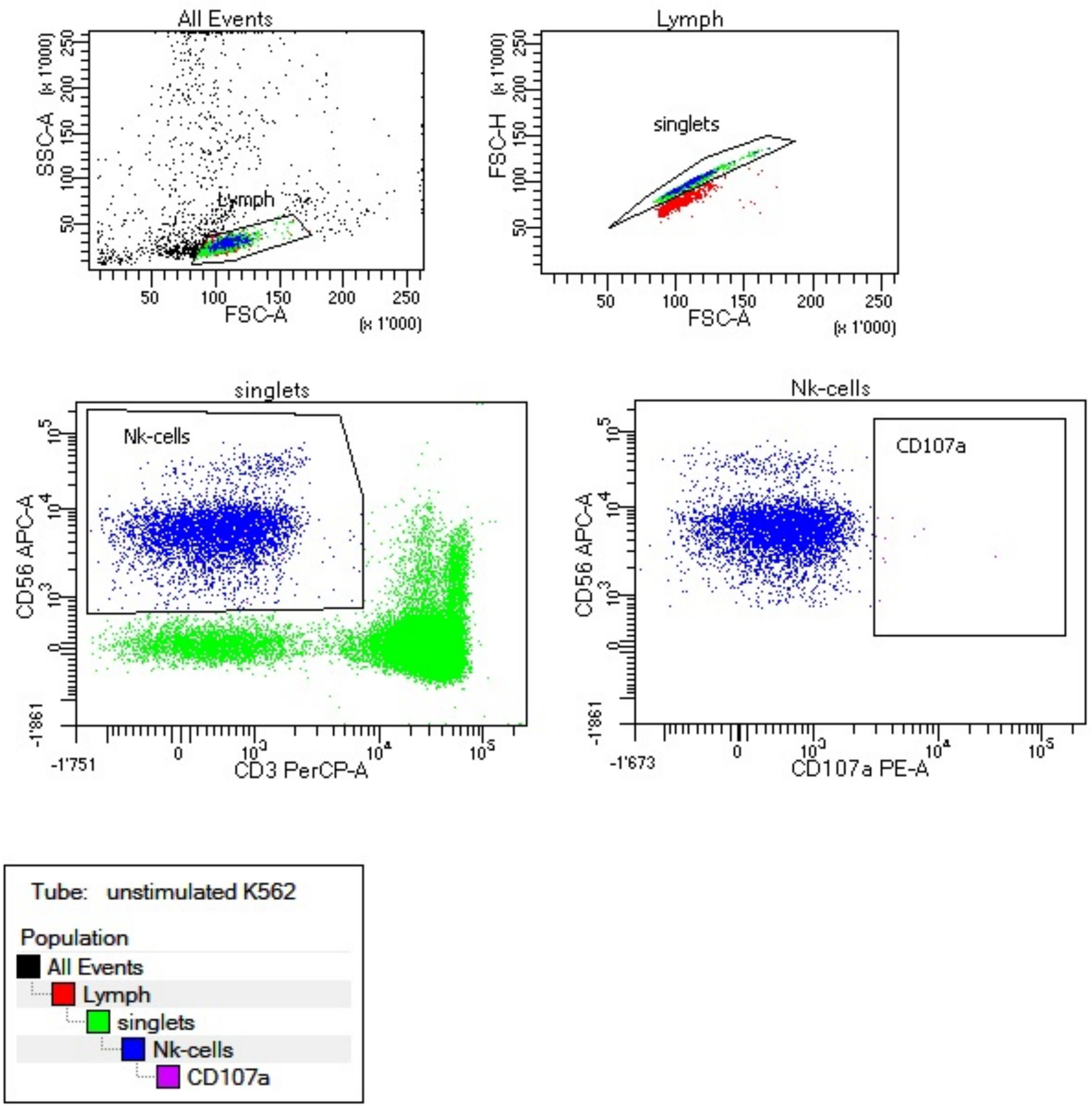

Experiment Name:	Degranulation_O_20230523	Tube Name:	unstimulated K562
Specimen Name:	Degranulation Assay	Record Date:	23.05.2023 17:00:25

Population	#Events	%Parent
All Events	82'981	####
Lymph	63'009	75.9
singlets	55'979	88.8
Nk-cells	4'976	8.9
CD107a	11	0.2

Supplementary Figure 1. Gating strategy of the NK cell degranulation assay. The gates are set for the PBMC sample before K562 incubation.

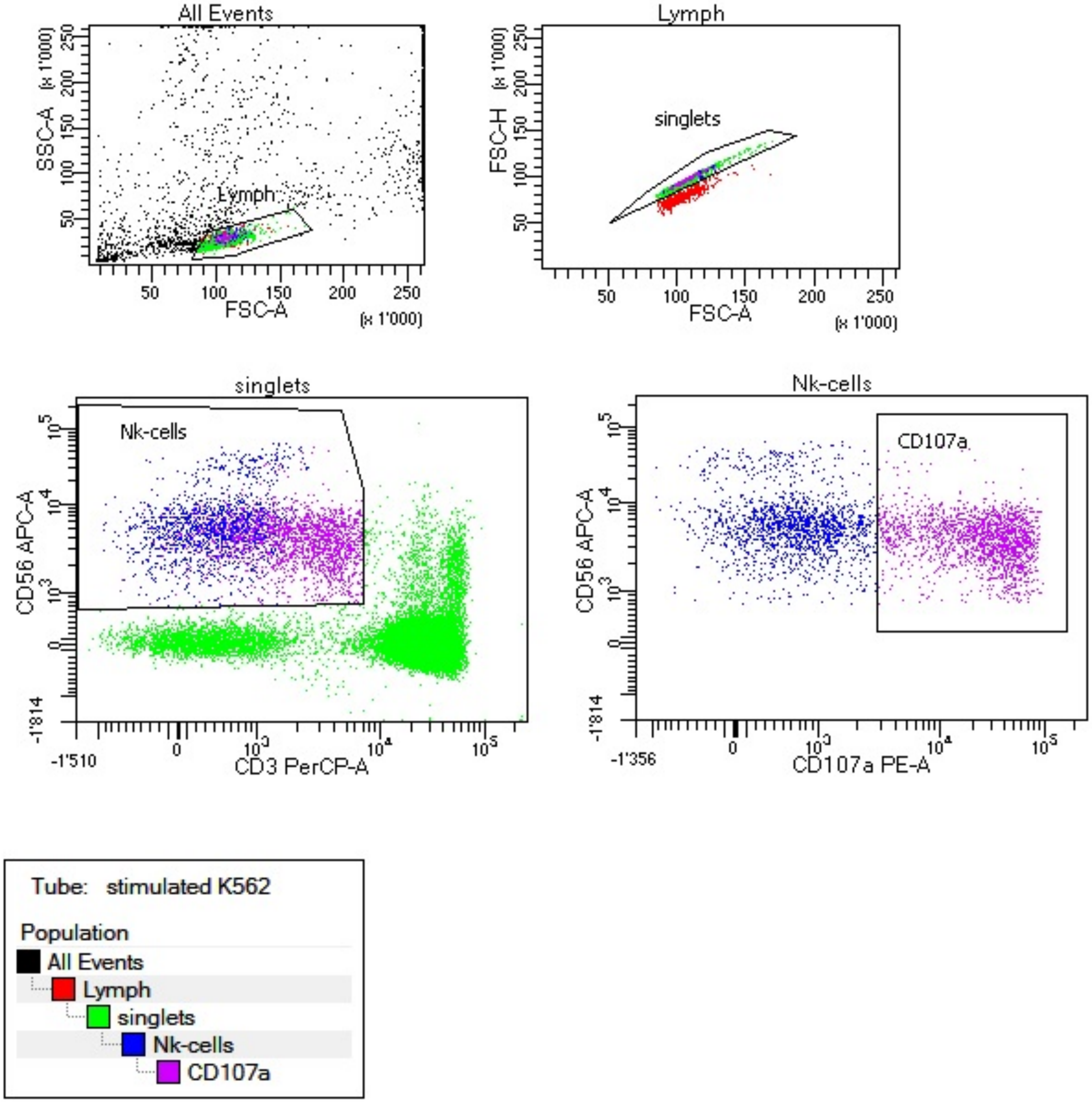

Experiment Name:	Degranulation_O_20230523	Tube Name:	stimulated K562
Specimen Name:	Degranulation Assay	Record Date:	23.05.2023 17:02:18

Population	#Events	%Parent
All Events	83'895	####
Lymph	45'219	53.9
singlets	39'311	86.9
Nk-cells	3'170	8.1
CD107a	1'645	51.9

Supplementary Figure 2. Interpretation of the NK cell degranulation assay after K562 incubation. Events on flow cytometer analysis are sufficient (>250). Degranulation is indicated through the %CD107a expression (51.9%). Here is an example of a sample without a degranulation deficiency.

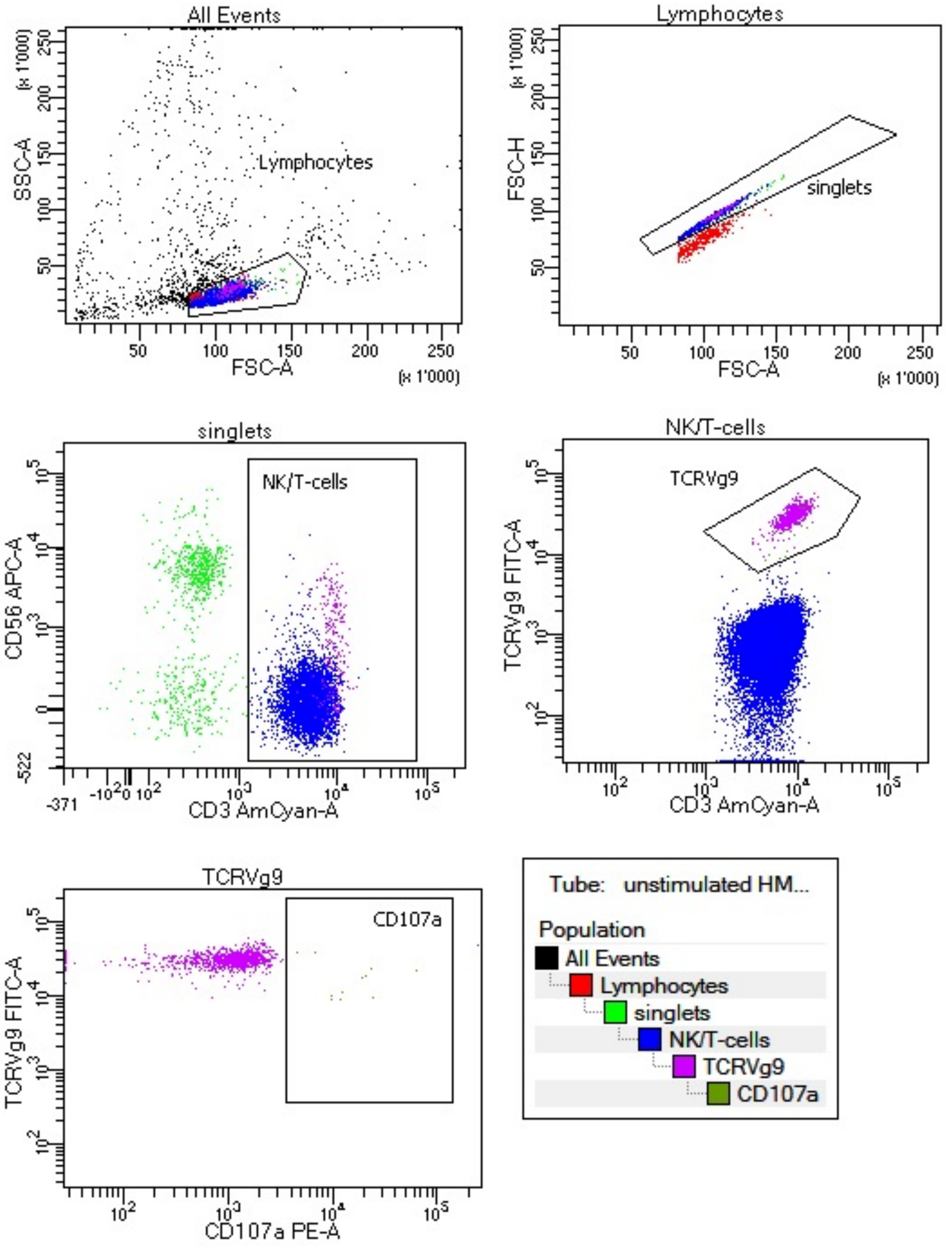

Experiment Name:	Degranulation_O_20230523	Tube Name:	unstimulated HMBPP
Specimen Name:	Degranulation Assay	Record Date:	23.05.2023 17:03:05

Population	#Events	%Parent
All Events	39'955	####
Lymphocytes	30'557	76.5
singlets	26'914	88.1
NK/T-cells	23'301	86.6
TCRVg9	1'016	4.4
CD107a	11	1.1

Supplementary Figure 3. Gating strategy of the Vγ9Vδ2 T cell degranulation assay. The gates are set for the PBMC sample before HMBPP stimulation.

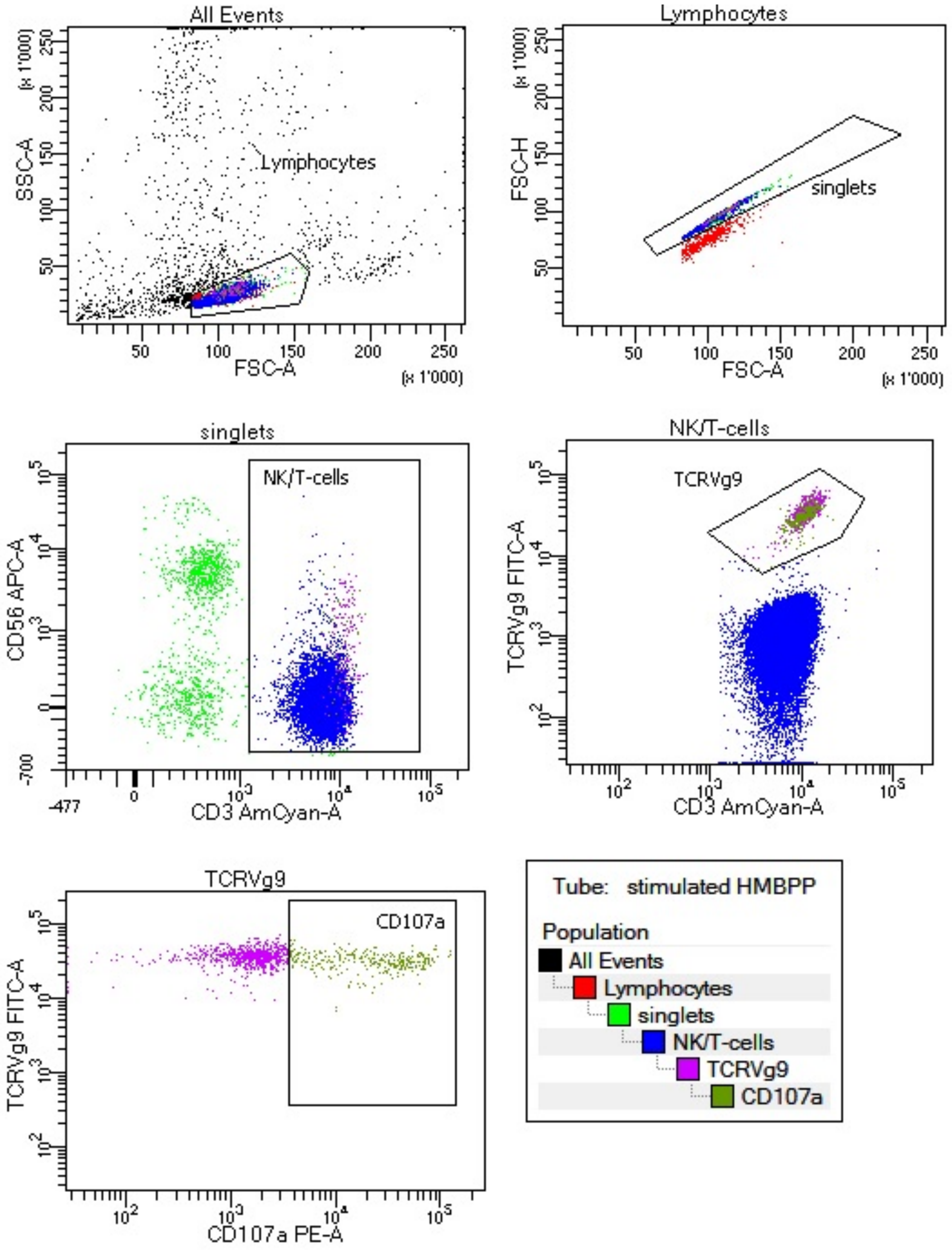

Experiment Name:	Degranulation_O_20230523	Tube Name:	stimulated HMBPP
Specimen Name:	Degranulation Assay	Record Date:	23.05.2023 17:03:52

Population	#Events	%Parent
All Events	53'920	####
Lymphocytes	41'117	76.3
singlets	36'458	88.7
NK/T-cells	30'915	84.8
TCRVg9	1'014	3.3
CD107a	297	29.3

Supplementary Figure 4. Interpretation of the Vγ9Vδ2 T cell degranulation assay after HMBPP stimulation. Degranulation is indicated through the %CD107a expression (29.3%), events are sufficient (>250). Here is an example of a sample without a degranulation deficiency.

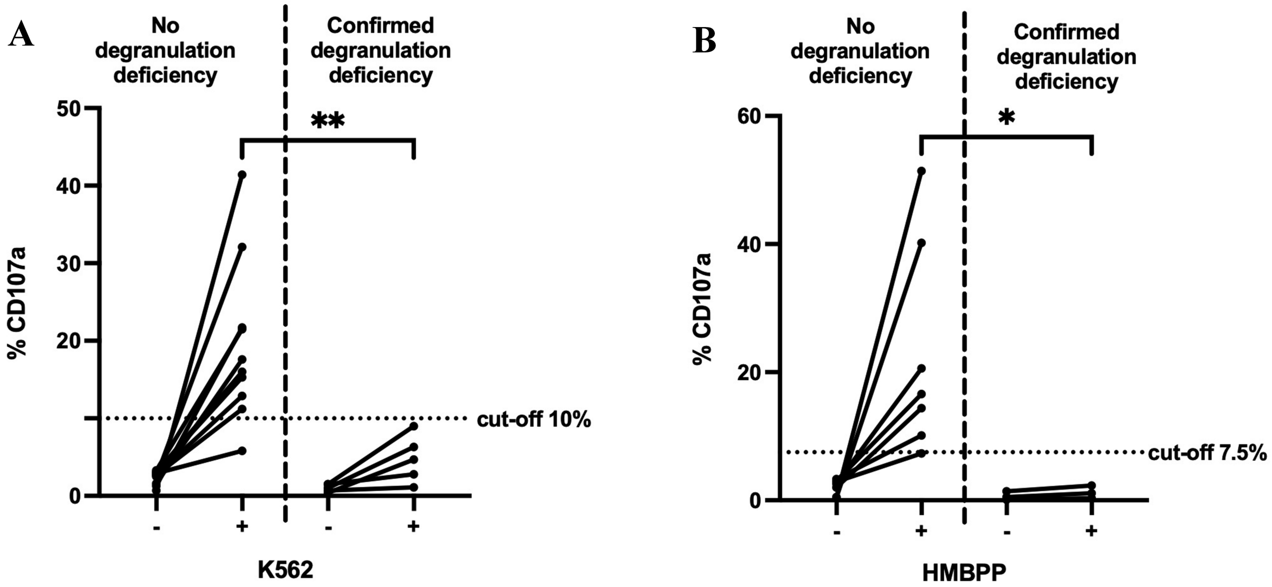

Supplementary Figure 5. NK cells and Vγ9Vδ2 T cells from cryopreserved PBMCs can degranulate. (A) The NK cell degranulation assay and (B) the Vγ9Vδ2 T cell degranulation assay performed with cryopreserved PBMCs (n=15) before (-) and after (+) stimulation with K562 cells and HMBPP, respectively. The samples were retrospectively divided into a control group (individuals without any known degranulation deficiency; on the left side of each graph) and an fHLH group (individuals with confirmed degranulation deficiency; on the right side of each graph). **p=0.0027 and *p=0.0167 in a Mann-Whitney test.
